# Public health role in litigation to address climate change

**DOI:** 10.1093/eurpub/ckad221

**Published:** 2024-01-12

**Authors:** Giovanna Mazzola, David Patterson, Farhang Tahzib

**Affiliations:** Environmental Health Working Group, World Federation of Public Health Associations, Geneva, Switzerland; Department of Transboundary Legal Studies, Faculty of Law, Rijksuniversiteit Groningen, Groningen, Netherlands; Public Health Ethics Committee, Faculty of Public Health, London, UK

In 2023, the Grand Chamber of the European Court of Human Rights commenced hearing its first climate case, KlimaSeniorinnen v. Switzerland, which deals with the impact of climate change on the elderly.^1^ Public health expertise to provide evidence of the health impacts of climate change in Europe is increasingly sought for such litigation. In November 2023, the European Public Health Association (EUPHA), the Faculty of Public Health (UK), the Aletta Jacobs School of Public Health (Netherlands) and other partners launched *From Analysis to Action: Climate Change Litigation. A Guide for Public Health Professionals*.^2^

The past several years have seen a significant rise in litigation over actions or inaction related to climate change. The number of climate change court cases has more than doubled since 2017 and is growing worldwide.^3^ While most cases have been brought in the USA, Europe, the UK and Australia, ∼17% of cases are now being reported in developing countries, including Small Island Developing States.

By using litigation to oblige governments and private sector polluters to address the climate crisis, claimants have been pushing for declaratory judgements and court orders requiring governments or private sector polluters to take specific action or stop an ongoing course of action. Moreover, climate change litigation creates opportunities for public scrutiny and debate, raising awareness around governments’ inaction or harm caused by private sector polluters.

One of the main legal hurdles faced in climate change litigation is proving the direct or indirect linkage between the (in)action and the subsequent harm to claimants. Expert testimony proves to be decisive: through research and oral or written testimony, public health experts provide a compelling narrative that allows the court to establish a linkage between the (in)action challenged and the evidence of past, present or future health harms. Consequently, strong cooperation between the legal and the scientific communities is essential to the successful outcome of climate change litigation. Yet, public health professionals and scientists are often unaware of the opportunities offered by litigation to address climate change related health issues and of their crucial role in collecting, securing and presenting evidence of harms to human health.^4^

The Guide is thus intended to introduce public health professionals to the concept of climate change litigation and explain how their expertise can contribute to making a legal case against private sector polluters or intransigent governments. Its content draws on suggestions made by World Federation of Public Health Associations (WFPHA) member organizations and public health experts through an on-line survey of representatives of national public health associations of WFPHA member countries and other public health professionals. The survey assessed the level of engagement and the interest shared by respondents in climate change issues and their current level of cooperation with local legal expertise.

Overall, the survey demonstrated that there is a keen interest in the public health community to better understand the importance of climate change litigation in addressing climate-related health issues and the opportunities offered by interdisciplinary collaboration. Of critical interest was the finding that while the majority of respondents (79%) reported they are already working on issues related to climate change, many of them (57%) do not have ready access to legal expertise, either internal or external, for guidance on legal and policy matters.

Respondents were also asked how interdisciplinary collaboration to address climate change could be strengthened. Most recommended joint seminars, workshops and conferences to compare perspectives, build a common language and share legal and scientific tools. These occasions could also serve to raise public awareness about the impact of climate change on health and the role played by climate change litigation in addressing related violations. In addition, some respondents suggested guidelines on climate change litigation to train future generations of public health professionals, and disseminating knowledge about the legal tools to address the impact of climate change on human rights.

With much of the decision-making in many countries going in the wrong direction to counter climate change, new initiatives are needed to make things happen. Litigation is one initiative taking momentum, and the Guide may help public health professionals to navigate in this field.^5^

## Data Availability

The data underlying this article will be shared on reasonable request to the corresponding author.
